# Assessment of chronic kidney disease using skin texture as a key parameter: for South Indian population

**DOI:** 10.1049/htl.2016.0098

**Published:** 2017-05-23

**Authors:** Madhanlal Udhayarasu, Kalpana Ramakrishnan, Soundararajan Periasamy

**Affiliations:** 1 Department of Biomedical Engineering, Rajalakshmi Engineering College, Chennai, India; 2 Department of Nephrology, Sri Ramachandra University, Chennai, India

**Keywords:** diseases, skin, biological organs, neural nets, health care, chronic kidney disease, skin texture, South Indian population, glomerular filtration rate, CKD epidemiology collaboration equations, artificial neural network

## Abstract

Periodical monitoring of renal function, specifically for subjects with history of diabetic or hypertension would prevent them from entering into chronic kidney disease (CKD) condition. The recent increase in numbers may be due to food habits or lack of physical exercise, necessitates a rapid kidney function monitoring system. Presently, it is determined by evaluating glomerular filtration rate (GFR) that is mainly dependent on serum creatinine value and demographic parameters and ethnic value. Attempted here is to develop ethnic parameter based on skin texture for every individual. This value when used in GFR computation, the results are much agreeable with GFR obtained through standard modification of diet in renal disease and CKD epidemiology collaboration equations. Once correlation between CKD and skin texture is established, classification tool using artificial neural network is built to categorise CKD level based on demographic values and parameter obtained through skin texture (without using creatinine). This network when tested gives almost at par results with the network that is trained with demographic and creatinine values. The results of this Letter demonstrate the possibility of non-invasively determining kidney function and hence for making a device that would readily assess the kidney function even at home.

## 1 Introduction

Glomeruli are the tiny filter in the kidney that filters out the waste from blood. The effectiveness of these filtrations determines the functionality of the kidney. Glomerular filtration rate (GFR) is one effective parameter that estimates the performance of glomeruli and hence the kidney (Peake *et al.*). There are many standard algorithms such as modification of diet in renal disease (MDRD) equation, chronic kidney disease (CKD) epidemiology collaboration (CKD-EPI) etc. to compute GFR using biochemical indices such as serum creatinine (Scr) and demographic characteristics such as age, gender, height, weight etc. However accurate filtration rate could be measured using nuclear technique by injecting isotopes such as, (_25_I) iothalamate, 99mTcdiethylenetriaminepentaacetic acid and iohexol [1]. Since, these markers are radioactive by nature and also expensive, this kind of GFR computation is not used unless the situation warrants. Therefore, equation-based methods are used in common for GFR computation.

Scr is a protein in the blood that normally elevates on renal dysfunction (Andrew *et al.*, 2004). However, its elevation may also be due to ingestion of cooked meat or increased intake of protein or even any infection in kidney. Therefore, gauging kidney function based on GFR is better than that with only Scr. MDRD method of GFR computation is one widely used and undergoing rapid modification, specifically based on ethnic values [2]. Uwe *et al.* [3] made a study on renal transplant recipients and concluded that calculation of GFR by the Mayo Clinic equation and the Rule's refitted-MDRD formula led to improved diagnostic performance in renal transplant recipients.

GFR value is also influenced by food [2] and plasma clearance (Pcr) [4]. Ma *et al.* [5] included cystanine C with Pcr for estimating GFR and found to be better than modified MDRD equation. Cockcroft–Gualt (CG) estimated GFR based on creatinine cr (Scr). After the introduction of MDRD and CKD-EPI equations, the supremacy of CG equation has slightly reduced [6]. Ary *et al.* [7] says MDRD and CG equations are imprecise for obese patients and Salazar–Corcoran equation is often proved to be unbiased estimate. Matsushita *et al.* have concluded that CKD-EPI method of GFR computation is more accurate than modified MDRD equation in acute decompensated heart failure. These equations use ethnicity as one of its parameter that quantifies race just in two values, black and white. However, in practise there are subjects which cannot be exactly classified into only two levels, and therefore this leads to approximation and error.

Many studies in the field of assessing ethnic parameter are carried for people belonging to China, Japan, USA and Africa [2, 8]. All these studies again boils down to only two values, one for black and the other for white. To our knowledge no such study is carried out in India, specifically for South Indian population where the people will not come under either of this group. Therefore, attempted in this Letter is to find an equivalent value to ethnic parameter for every individual depending on their skin texture and evaluating GFR based on this value. To perform this study, first requirement is relevant real-time data. Hence, demographic and biochemical indices were collected from volunteers of normal and CKD group. New GFR equation is arrived and CKD levels were determined using only demographic and skin parameters. The results obtained are agreeable with clinical results.

## 2 Data collection

The standard demographic characteristics and the biochemical indices are obtained from the patient in the age group between 14 and 75 years (male and female), the mean value of the age is 43.46 and the standard deviation is 17.86. In this, 64 subjects belong to CKD and 11 subjects to normal category. All these subjects are from different states belonging to South India. Necessary permission and individual consent were obtained as per the institutional policy. Tried herein is to quantify ethnic parameter based on skin tone and therefore skin images are also obtained from the entire subjects using Samsung Galaxy Tab A with the resolution of 2592 × 1944 (5 megapixels) and 1 pixel representing an area of 20.83 × 36 µm^2^. The images are acquired at normal room temperature with standard lightning arrangement with no external interference. All the images are acquired from the lateral position of the forehand.

## 3 GFR computation

The value of GFR indicates the stage of CKD, >90 for normal subjects and <90 in renal failure cases [9]. As the filtration process goes down the GFR values start decreasing indicating the failure of kidney function [10].There are some standard methods for estimating GFR such as; CG, MDRD, CKD-EPI, Jelliffe, Mawer, Bjornsson and Gates [1, 10–12]. Among this MDRD and CKD-EPI (as given below) are widely used that uses demographic characteristics and the biochemical indices [9, 13, 14] as its arguments

$$\eqalign{ {{\rm MDRD}\colon } & {175 \times S_{{\rm cr}}^{ - 1.154} \times \hbox{ag}\hbox{e}^{ - 0.203}{\rm for}\, {\rm men}} \cr & {175 \times S_{{\rm cr}}^{ - 1.154} \times \hbox{ag}\hbox{e}^{ - 0.203} \times 0.742\, \left({{\rm if}\, {\rm female}} \right)\times 1.212\, \left({{\rm for}\, {\rm blacks}} \right)} \hfill \cr {{\rm CKD - EPI}\colon } & {141 \times {\rm min}{\left({S_{{\rm cr}}/k\comma \; \, 1} \right)}^\alpha \times {\rm max}{\left({S_{{\rm cr}}/k\comma \; \, 1} \right)}^{ - 1.209} \times {0.993}^{{\rm age}}} \cr & { \times 0.018\left({{\rm female}} \right)\times 1.159\, \left({{\rm black}} \right)} } $$

*k* = 0.7 female, *k* = 0.9 male, *α* = − 0.329 female and *α* = − 0.411 male.

Presently, GFR computation algorithm involves four parameters out of which ethnic value is one that has been evaluated for western region of the globe (either black or white). However, the race of South Indian population does not come under this category, since their skin tone is different. It would be better to have a separate ethnic value for this section of people to analyse their renal functionality. Therefore, attempted here is to compute ethnic value of people who do not come under black or white category (typically South Indian inhabitants) through skin texture.

### 3.1 Analysis of skin texture

Skin wrinkles are common occurrence that is formed due to various factors such as the loss of skin elasticity, skin dryness, skin thinning and ageing. Skin care is crucial in preventing skin breakdown and venous ulcers especially during kidney dysfunction [15]. Sometimes permanent deformation also sets in skin when the remodelling capability of collagen [16] is collapsed, as collagen is the one that binds muscles and bones with skin. The exterior manifestation of muscular changes, hydration [17] and wrinkles contribute to skin texture when viewed through a digital camera. Since these parameters change with the level of CKD [18] (water content of the body changes [19] and the muscle breakdown protein creatinine increases [20]), it could be said that CKD stage would have impact on skin texture. Assessment of such skin textural characteristic has got much clinical interest, to identify underlying physiological condition. In this process, since skin texture has a close relationship with hydration [17], the texture of the skin should vary with the level of kidney disease. To establish this relation, textural analysis is carried out on the skin images obtained through digital camera from the lateral view of forehand of all the subjects under study.

**Fig. 1:**
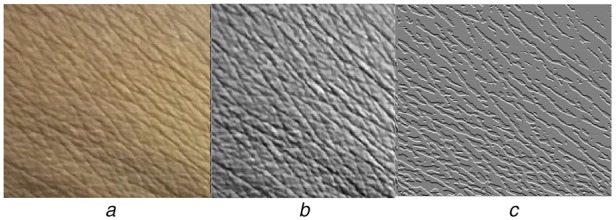
Skin images of CKD subjects *a* Original image *b* Enhanced image *c* Directional gradient

**Fig. 2:**
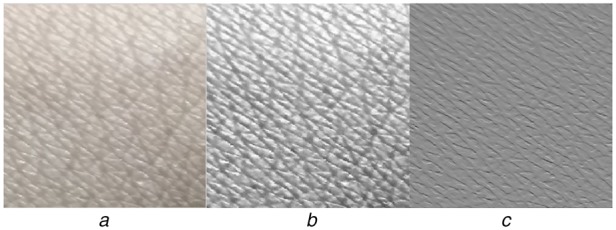
Skin images of normal subjects *a* Original image *b* Enhanced image *c* Directional gradient

These images show the wrinkles as line like formation. These lines appear in both the directions and exhibits zigzag façade. Damanpreet and Prabhneet have analysed skin texture to identify skin disease. They have captured skin images using ultraviolet camera, where the skin image appears an array of closed structure because of the wrinkles that are exhibited as line like formation. This is what is also observed in our study, Fig. 2*a* is the image acquired from subjects without kidney dysfunction. On the other hand, the skin image obtained from CKD subject does show the closed structure, but exhibit more dense and intense lines as shown in Fig. 1*a*.

This kind of skin changes due to wrinkle formation in normal and CKD subjects is observed in all the 75 subjects considered in this Letter. To correlate the level of changes in skin with CKD stages, skin texture needs to be quantified. In general, image texture gives information about the spatial arrangement of grey-level intensity in the image [21] which in turn represents the nature of the tissue. Since these properties are of interest, and to extract them the obtained skin images are subjected to the three sequence operation as stated below:

(i) The images are first enhanced using morphological algorithm [17] to view the hidden information in the raw image.

(ii) The formation of the wrinkle (appearance of the line in the image) is of interest in this Letter and is much orientation dependent, hence directional gradient for all the images (*f*) with respect to the *x*-axis and the *y*-axis are computed.

The gradient of the image is given by *gx* = ∂*f*/∂*x* along the *x*-direction and *gy* = ∂*f*/∂*y* along the *y*-direction.

The gradient direction *θ* can be calculated by $\tan ^{ - 1}\left[{\lpar gy/gx\rpar } \right]$The sample of acquired image which was processed by for enhancement and directional gradient is presented in Figs. 1 and 2 for CKD subjects and normal subjects, respectively.

(iii) The directional gradient image explicitly visualises the line formation due to wrinkles. These wrinkles are one major factor that changes the skin texture in CKD subjects [22] and hence texture analysis is performed over the processed image. Statistical characterisation of images would give information pertaining to intensity distribution and its correlation. However, to analyse skin texture and to correlate with CKD level second-order statistical approach could give better information involving distance and orientation of the pixel. Therefore, grey-level co-occurrence matrix (GLCM) is computed for the processed image. GLCM quantifiably describes the associated grey-level variation in the image. From the computed matrix, statistical features are obtained which are the representations of skin changes due to physiological changes including kidney functions. If such statistical attributes of image intensity are extracted, then they could be used as metrics for classifying the observed changes in skin morphology with kidney function. In summary, for an image with different grey-level intensity, the corresponding GLCM would be a tabulation of how often different combination of occurrences of grey-level of pixel in an image or an image section [23, 24]. From GLCM, Haralick's parameter [25] that is the image property could be extracted. Haralick *et al.* proposed 14 features that could be deduced from GLCM. However, out of this only four parameters are used in this Letter:

- *Contrast:* Measures the amount of local variation present in the image.
- *Energy:* It signifies the distance of grey-level intensity in periodic form or otherwise (uniformity).
- *Correlation:* It measures the linear dependency of grey-level values in GLCM.
- *Entropy:* It measures the randomness or disorderness of the image.

### 3.2 Skin texture as random process

**Fig. 3:**
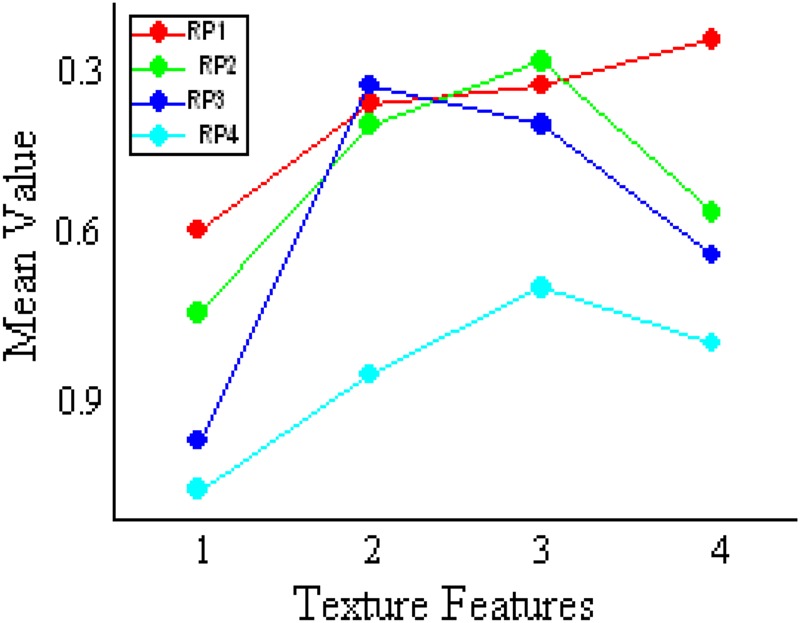
Mean of texture values for different random processors (1 – contrast, 2 – correlation, 3 – energy and 4 – homogeneity)

Formation of skin texture is the result of many physiological processes such as ageing and pathological condition. Since this process is influenced by many factors and its occurrence is not predictable, also, in general, texture is a random process (RP) [26], this (skin texture) can be considered as an RP. In this Letter, the clinical data obtained is grouped into five different levels, starting from level 1 as normal to level 5 as the highest stage of CKD. Out of 75 cases, 11 are in level 1 and the remaining 64 are in CKD stage (16 belonging to each level). On the basis of the disease condition, these five levels are considered as five different RPs. These are the ensemble of one RP, that is, skin texture. In general, an RP consists of ensemble of random variables (RVs). Similarly, these processes which are characterised by its textural features are its RVs. Hence, each RP now has collection of RV such as contrast, energy, correlation and homogeneity. Mean value of these RVs for every RP is computed and plotted in Fig. 3. Ensemble averages over the mean of RVs for every RPs are given in Table 1.

**Table 1:** Mean of random processors

RP	Level	Mean of process	Deviation from level 1, %
RP1	level 1	0.835 ± 0.012	—
RP2	level 2	0.80 ± 0.019	4.19
RP3	level 3	0.6903 ± 0.02	17.329
RP4	level 4	0.6058 ± 0.025	27.449
RP5	level 5	0.3508 ± 0.034	57.98

From Table 1, it can be observed that the deviation in mean of the processors is significant as the CKD stage advances. Since this mean value gives significant difference, it is considered as one of the parameters for developing classification system.

### 3.3 Four parameter (4P) model

These four features are the indicators of individual's skin texture. However, to determine the relationship between these four textural parameters and CKD levels or GFR, a non-linear regression model would be useful. Hence, 4P model (4PM) is adopted

(1) $$S_{\rm p}\left({x\left({a\comma \; \, b\comma \; \, c\comma \; \, d} \right)} \right)= d + \left({a - d} \right)/\lpar 1 + \left({x/c} \right)^b\rpar \eqno\lpar 1\rpar $$

**Fig. 4:**
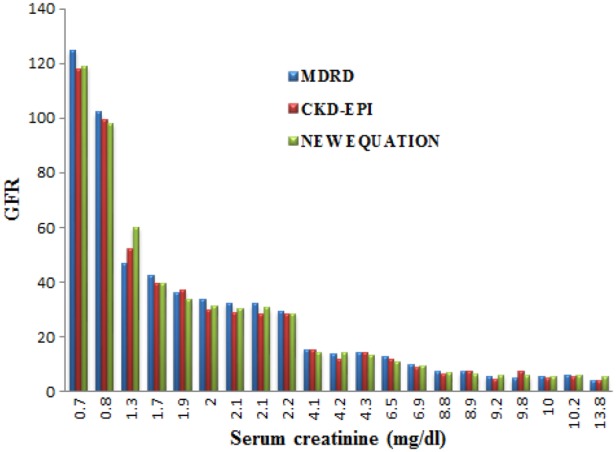
GFR obtained through MDRD, CKD-EPI, proposed method for a sample of data

In general, this model is used for curve fitting procedure, done for every point on the *x*-axis. However, it is used here to obtain a unique measure for every individual describing his/her skin texture. The arguments *a*, *b*, *c* and *d* in this model (1), respectively, represent minimum asymptote, hill slope, infection point and maximum asymptote. When the mean values of the four textural parameters are plotted (as shown in Fig. 4), it could be observed that homogeneity takes the highest value and contrast takes the lowest value, hence they are taken here as the parameter ‘*d*’ and ‘*a*’. Similarly, parameter ‘*b*’ is energy, ‘*c*’ is correlation and ‘*x*’ is age. Since these four textural parameters varies for every individual (marked by age), a fit point is computed by taking age as the argument ‘*x*’ in the model. Since these four textural parameters vary for every individual (marked by age), a fit point is computed by taking age as the argument ‘*x*’ in the model. With this, a new metric *S*_p_(*x*(*a*, *b*, *c*, *d*)) is now computed.

Glomerular filtration is one primary step in separating the waste product from the blood; any alteration kidney function will have a direct impact on filtration rate. Therefore, GFR is one widely accepted measure to assess the kidney function. However, there are lot of algorithms to compute GFR, most of the algorithms use one of its parameter describing ethnic characteristics with two values (one for white and other for black). Instead of grouping different subjects into only two groups and assigning a constant for each group as ethnic value, it would be better to have a numerically quantified value suitable for every individual. This would result in still more accurate GFR. This is what is accomplished through the result of 4PM, which is now replaced in GFR equation in place of ethnic value The proposed GFR is given in (2)

(2) $${\rm GFR} = 175 \times S_{{\rm cr}}^{ - 1.154} \times \hbox{ag}\hbox{e}^{ - 0.203} \times S_{\rm p}\left({x\left({a\comma \; \, b\comma \; \, c\comma \; \, d} \right)} \right)\eqno\lpar 2\rpar $$

GFR value computed through the above-mentioned algorithm is agreeable when compared with the MDRD, CKD-EPI algorithm. The variation of GFR obtained through these three procedures (proposed, MDRD and CKD-EPI) against creatinine is plotted in Fig. 4.

## 4 Classification

In the previous section, it was proved that the GFR value obtained using skin parameter *S*_p_ was comparable with values obtained using traditional methods. In this section, artificial neural network (ANN) is used to build a decision support system to determine the kidney function based on demographic values and skin parameter obtained through (1) without using creatinine value. ANN has strong interconnection of neurons to perform set of sequential operation. In general, it is a mathematical model applied for classification and decision making application including biomedical applications [27, 28]. In this work, feed-forward network is adopted to categorise the CKD level based on demographic parameters. Normally, CKD level is determined from GFR value as described below [14]

- If GFR ≥ 90 = normal or elevated GFR – level 1.
- If GFR 60–89 = mild GFR reduction – level 2.
- If GFR 30–59 = moderate GFR reduction – level 3.
- If GFR 15–29 = severe GFR reduction – level 4.
- If GFR < 15 = renal failure – level 5.

**Fig. 5:**
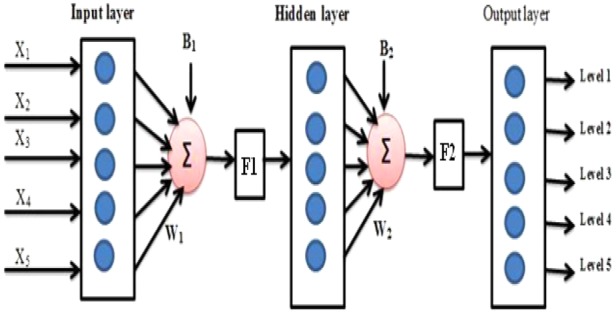
Architecture of neural network used for classification

Two types of feed-forward network are constructed: first one consists of five input neurons, one hidden layer with five neurons and the output layer with five neurons as illustrated in Fig. 5. It takes five inputs such as age (years), gender (1 – male, 2 – female), height (centimetres), weight (kilograms) and Scr (milligram/decilitre). The second network is constructed with seven input neurons, one hidden layer with five neurons and the output layer with five neurons as illustrated in Fig. 5. It takes seven inputs such as age (years), gender (1 – male, 2 – female), height (centimetres), weight (kilograms), texture parameter *S*_p_ and mean of texture features.

All the five neurons in the output are meant for displaying CKD level, i.e. if the output is 10,000, then classified as CKD level 1 and if 01000, then CKD level 2 and so on. The sigmoidal training function (F1) is used between input layer and hidden layer and linear function (F2) is used between hidden and output layers.

The neurons in the input layer receive the data and transfer them to neurons in the hidden layers and the output of hidden layer (*y_j_*) is transferred to output layer through the weighted links as shown in (3) and (4)

(3) $${\bi y}_j = {\rm F}1\left({\left[{\sum\limits_t {\sum\limits_{\,j = 1}^s {x_t{\bi W}_{\!ij}} } } \right]+ b_j} \right)\eqno\lpar 3\rpar $$

(4) $${\rm output}\, {\rm at}\, {\rm level}\, k = {\rm F}2\left({\left[{\sum\limits_t {\sum\limits_{\,j = 1}^s {y_j{\bi W}_{\!jk}} } } \right]+ B_k} \right)\eqno\lpar 4\rpar $$

where *i* varies between 1 and 5 for network 1 and between 1 and 7 for network 2, *j* and *k* varies between 1 and 5, ***W****_ij_* is the weight matrix between input and hidden layers, ***W****_jk_* is the weight matrix between hidden and output layers, ***b*** and ***B*** are the respective bias to hidden and output neurons and levels 1–5 are different outputs indicating CKD stage.

A database is created with eight columns such as age, gender, height, weight, Scr, skin texture parameter, mean of textural features and CKD level and 75 rows (records) belonging to different subjects. First network is trained with 45 records, input being columns 1–5 with known target as in column 8. Then, the second network that is again trained with 45 records, input being columns 1–4, 6 and 7 with known teacher value in column 8. Now, these networks are tested using remaining 30 records (of which 5 are not diagnosed for CKD) with the same respective inputs. The result of the two networks is presented in Table 2.

**Table 2:** Classification accuracy as obtained through the two networks constructed

Classification result	Network 1, %	Network 2, %
false positive	0	3.33
false negative	3.33	3.33
true positive	80	76.66
true negative	16.6	16.66

## 5 Conclusion

**Fig. 6:**
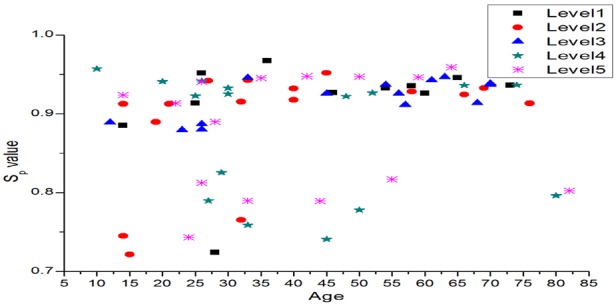
Distribution of P-value with age and CKD level

Attempted in this work is to non-invasively assess the kidney function. At present, the firsthand information is obtained through GFR. Widely used algorithms such as MDRD, CKD-EPI for GFR computation is designed for whites and blacks, few research are seen in Japan and China. To our knowledge, no ethnic evaluation is carried out for people who are neither black nor white, typically South Indian population. With the fact that CKD could influence skin, and therefore envisaging skin texture is one possible way of assessing a metric equivalent to ethnic value, skin images from lateral part of forehand are captured from control and CKD subjects at standard environment conditions. From the textural features (that are due to the manifestation of internal composition), a single value that could indicate the racial aspect or ethnic value for South Indian population is computed using 4PM. The resulting value (skin parameter) which when substituted in standard MDRD equation, in place of ethnic value, GFR value is agreeable with the values obtained using standard procedure. The variation of *S*_p_ value with age and skin tone is plotted in Fig. 6. It can be found that, for the data we collected, *S*_p_ value lies between 0.65 and 1.00. It can also be found that, for relatively dark skinned subjects, this value is found to lie between 0.65 and 0.85, but for other subjects it is between 0.88 and 1.00. This demonstrates that *S*_p_ value, so called ethnic parameter for south Indian population widely lies in the spectrum of 0.88–1.00.

As discussed in Section 3.3, each RP consists of four texture features (as RV) per subject. The mean over RV for every individual under each process is almost constant as tabulated in Table 1. This confirms that average textural response of the skin is almost similar under every category/CKD level. Furthermore, since this mean value gives significant difference among the processes, it becomes one of the valuable parameter that could be used while designing a classification machine (here ANN).

**Fig. 7:**
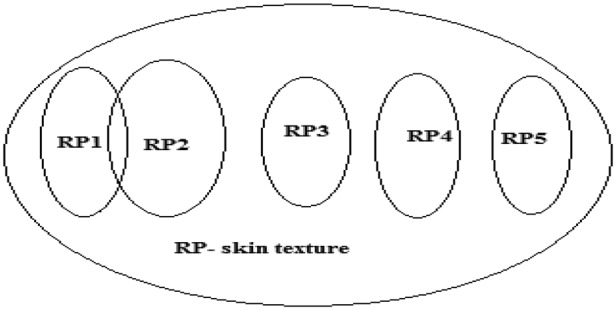
Skin texture as ensemble of RPs

However, RP1 and RP2 overlaps over a small range as shown in Fig. 7 and Table 1, respectively, which is the reason for misclassification (false positive and false negative) as given in Table 2. Apart from this all the classes are quite separable and give right classification. From the above discussion, it is concluded that the result of this Letter will be useful for developing a device that could non-invasively assess kidney function that could be such as screening test. This device which when developed would be much beneficial to the society, specifically in rural areas. At present, kidney dysfunction is observed mostly after level 3, where treatment becomes difficult and chances of entering into dialysis or kidney transplantation stage are high. In this context, the proposed work (non-invasive technique) would facilitate for periodical monitoring and hence enable earlier diagnosis.

## 6 Funding and declaration of interests

Conflict of interest: none declared.
